# The Role of Computational Fluid Dynamics in the Management of Unruptured Intracranial Aneurysms: A Clinicians' View

**DOI:** 10.1155/2009/760364

**Published:** 2009-08-19

**Authors:** Pankaj K. Singh, Alberto Marzo, Stuart C. Coley, Guntram Berti, Philippe Bijlenga, Patricia V. Lawford, Mari-Cruz Villa-Uriol, Daniel A. Rufenacht, Keith M. McCormack, Alejandro Frangi, Umang J. Patel, D. Rodney Hose

**Affiliations:** ^1^Departments of Medical Physics and Neurosurgery, Royal Hallamshire Hospital, Sheffield, UK; ^2^Academic Unit of Medical Physics, School of Medicine and Biomedical Sciences, University of Sheffield, Sheffield, UK; ^3^Department of Neuroradiology, Royal Hallamshire Hospital, Sheffield, UK; ^4^NEC Laboratories Europe, NEC Europe Ltd., 53757 St. Augustin, Germany; ^5^Clinic of Neurosurgery, Department of Clinical Neurosciences, Geneva University Hospital, 1211 Geneva, Switzerland; ^6^Biomedicine Communication Technologies Department, Center for Computational Imaging & Simulation Technologies, Pompeu Fabra University, Barcelona, Spain; ^7^Department of Neuroradiology, Institute of Radiology, University Hospital Basel, Petersgraben, Basel, Switzerland; ^8^Department of Neurosurgery, Royal Hallamshire Hospital, Sheffield, UK

## Abstract

*Objective*. The importance of hemodynamics in the etiopathogenesis of intracranial aneurysms (IAs) is widely accepted. Computational fluid dynamics (CFD) is being used increasingly for hemodynamic predictions. However, alogn with the continuing development and validation of these tools, it is imperative to collect the opinion of the clinicians. *Methods*. A workshop on CFD was conducted during the European Society of Minimally Invasive Neurological Therapy (ESMINT) Teaching Course, Lisbon, Portugal. 36 delegates, mostly clinicians, performed supervised CFD analysis for an IA, using the @neuFuse software developed within the European project @neurIST. Feedback on the workshop was collected and analyzed. The performance was assessed on a scale of 1 to 4 and, compared with experts' performance. *Results*. Current dilemmas in the management of unruptured IAs remained the most important motivating factor to attend the workshop and majority of participants showed interest in participating in a multicentric trial. The participants achieved an average score of 2.52 (range 0–4) which was 63% (range 0–100%) of an expert user. *Conclusions*. Although participants showed a manifest interest in CFD, there was a clear lack of awareness concerning the role of hemodynamics in the etiopathogenesis of IAs and the use of CFD in this context. More efforts therefore are required to enhance understanding of the clinicians in the subject.

## 1. Introduction

The management of unruptured cerebral aneurysms remains one of the most controversial topics in neurosurgery. These uncertainties are multifactorial owing mainly to an incomplete understanding of the natural history of these lesions and risks associated with active management [1–4]. Recent evidence, however, suggests a good correlation between different hemodynamic factors and etiopathogenesis of IAs [5–8]. This, together with the fact that current technologies do not allow detailed in vivo measurements of blood flow [9, 10] in cerebral arteries has given computational fluid dynamics (CFD) new strength and a chance to affirm itself as a technology that can help in the management of unruptured IAs. Many studies have been published where patient-specific medical images and CFD are used to predict relevant hemodynamic variables that correlate well with initiation, growth and rupture of an IA [9–15]. Until recently, these analyses were performed primarily by engineers, physicists or mathematicians in collaboration with select clinicians. However, in order to make an impact on clinical practice and to enhance trust among clinicians, a controlled and extensive exposure of the software and its concepts to the broader clinical community is crucial along with its continuing validations. 

The current study is the first effort of its kind where the concept and application of CFD software was exposed to the clinical community, followed by analysis of their views, understanding and performance.

## 2. Material and Methods

The first gathering of the European Society of Minimally Invasive Neurological Therapy (ESMINT) Teaching Course on IAs provided an ideal opportunity to expose the computational tools being developed within the European project @neurIST [16], to the attention of its audience. The workshop was held near the birthplace of angiography at the “Edificio Egas Moniz” of the Hospital Santa Maria in Lisbon, Portugal between 7th and 12th September 2008.

### 2.1. Participants' Demography and Overview

**Figure 1:**
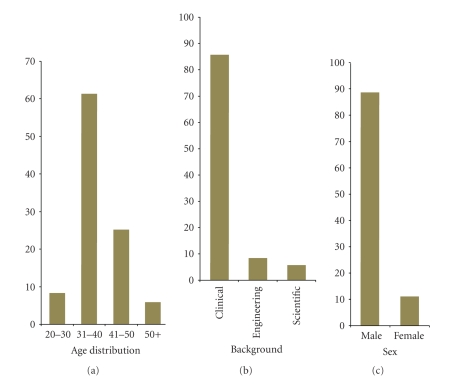
Participants' demographic constitution.

The workshop was attended mainly by neurosurgeons and neuroradiologists. Out of all participants 86% had a clinical, 8% an engineering, and 6% a scientific background. Participants broadly fell into four age groups: 20–30 years old (3 participants, 8%), 31–40 (22, 61%), 41–50 (9, 25%), 50+ (2, 6%). Participants were prevalently male with a ratio *M* : *F* = 8 : 1. These data are summarized in Figure 1. 

Participants were subdivided into groups of about 8 individuals per session to maximize teacher-to-attendee ratio. Two tutors were available during each session, one with a clinical background (neurosurgeon) and one with an engineering background (biomedical engineer). Teaching was delivered via a lecture of 75 minutes, which included a discussion of the clinical background and relevance of hemodynamic factors in etiopathogenesis of IAs, a brief introduction on the use of CFD in hemodynamic predictions, and explanation of key fluid dynamics concepts in this context, for example, wall shear stress (WSS), boundary conditions, and so forth. This was followed by a supervised hands-on experience with the software. 

**Figure 2:**
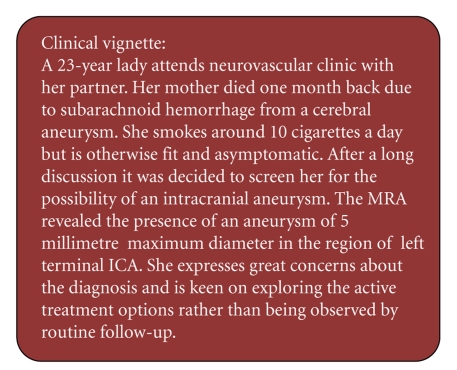
Clinical vignette: typical challenging case scenario.

The exercise was presented to the audience via a clinical vignette of a typical difficult scenario encountered in the clinic, represented in Figure 2. The vignette illustrates a typical case of an incidentally-discovered IA in an anxious young patient. Due to its size (5 mm maximum diameter) and absence of any other major known risk factors the aneurysm should be managed conservatively as per ISUIA (International Studies for Unruptured Intracranial Aneurysms) guidelines [1, 4]. However due to patient's concerns and insistence for active intervention the management plan becomes controversial.

The participants were then asked to use the software @neuFuse to extract additional and nonobservable hemodynamic data from the 3-dimensional rotational angiographic (3DRA) image of this case. Attendees performed the tasks independently with the help of a ready-reckoner containing the complete guided procedure with illustrations to facilitate the exercise. One-to-one support and supervision was provided during each session, as required.

### 2.2. Image Acquisition and Processing

The medical image used in the workshop was obtained using rotational acquisition in a Philips Integris Allura machine (Philips Medical Systems, Best, The Netherlands), producing 100 images in 6 seconds, with 5 ms exposure per image. Voxel size in the reconstructed 3D images was 234 microns with reconstruction matrix 256 × 256 × 256. Images were anonymized, respecting the @neurIST ethical approval for use of patient data. The characteristics of the aneurysm considered in this study are reported in Table 1.

The current version of the @neuFuse software (prototype 4), based on the Multimod Application Framework [17] and developed within the @neurIST project, was used to reconstruct the vessel surfaces, create the model and set up the hemodynamic analysis. The solvers used within @neuFuse to solve the fundamental equations describing the blood flow behavior within the region of interest were ANSYS-ICEM and ANSYS-CFX (Ansys, Inc., Canonsburg, PA, USA). 

For the purpose of the workshop a simple stationary analysis (nonpulsatile but constant flow rate and pressure at the openings of the region of interest) was performed by the participants using Intel core duo 2.4 GHz machines, with 2 GB RAM and 512 MB of dedicated graphic memory.

### 2.3. CFD Analysis

**Figure 3:**
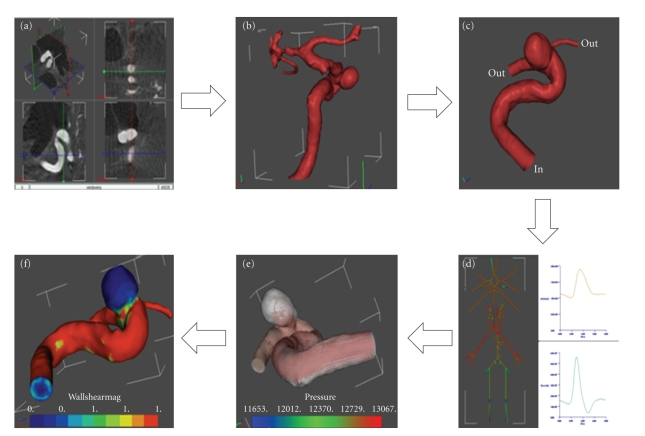
Operation workflow from medical image to hemodynamic results. (a) Orthoslice visualization of the 3DRA medical image in @neuFuse. (b) Visualization of the extracted vessel surface. (c) Visualization of reduced region of interest with location of inlet and outlet openings. (d) 1D circulation model. (e) Visualization of predicted streamlines. (f) Visualization of predicted wall shear stress.

Figure 3 shows the overall workflow of the operations performed from uploading the raw medical image to the software through the visualization of relevant hemodynamic data in @neuFuse. 

Any CFD analysis requires knowledge of the volumetric region traversed by the fluid (i.e., aneurysm including connected surrounding vasculature) plus information about velocity and pressure of the fluid at the boundaries of the chosen region of interest (boundary conditions). Participants were asked to reconstruct the region of interest starting from the medical image, and specify the boundary conditions using the software @neuFuse. The first step was to launch @neuFuse and import the medical image (Figure 3(a)). The geometry of the vessel, including the IA, was then extracted from the imported image (Figure 3(b)). As only a subregion of the extracted vasculature around the aneurysm has influence on the hemodynamic computation, all vessels entering or leaving the IA were identified and cropped at desired locations to define the region of interest (ROI). The ascending carotid artery, which was an inlet (blood enters the domain through it), was cropped at ten vessel diameters proximal to the IA as shown in Figure 3(c). The distal carotid artery in the region of cavernous sinus and the ophthalmic artery were identified as the two outlets of ROI (blood exits the domain through these two vessels). These are shown in Figure 3(c). For the sake of simplicity and time constrains the mesh used in the analysis was coarse and was constructed using simple tetrahedral elements. As is often the case in real-life clinical situations, information on pressure or velocity of the blood at these locations for the patient under examination was not available. Boundary conditions were therefore provided by using a 1D mathematical model of the systemic tree, which has been developed within @neurIST [18]. A representation of the @neurIST 1D circulation model is depicted in Figure 3(d). This model provides values of pressure and flow of blood at several locations along the systemic arterial tree, including locations in the circle of Willis for a typical individual. Plug-flow BCs were applied at inlet and pressure BC at outlet, using average values from the waveforms provided by the 1D circulation model. Typical values of blood viscosity (*μ* = 0.004 Pa*·*s) and density (*ρ* = 1066 kg/m^3^) were applied to define the blood properties. Although the blood is a nonNewtonian fluid for the sake of simplicity and time-constraints, and also in view of recent findings from Cebral et al. [13], we decided to assume constant blood viscosity. 

While arterial walls move under the effect of the propagating pressure waves, it has been shown that the effects of this movement on hemodynamic predictions are negligible [19, 20]. Fixed walls were thus considered in this analysis. The computation was automatically performed by the software and participants were asked to display different predicted hemodynamic variables like flow streamlines (Figure 3(e)), pressure distribution within the aneurysm or arterial wall, and WSS (Figure 3(f)). Participants were then asked to compare the WSS values computed within the aneurysm with the critical values found in the experimental studies of Malek et al. [21] below which the endothelium is affected by cell loss, desquamation and deranged activity of wall-growth regulators.

### 2.4. Evaluation

Finally, the feedback was collected via a questionnaire consisting of 48 questions. These were broadly divided into 6 categories (Table 2): general feedback, course design and conduct, experience with the software, hemodynamics understanding, impact of CFD in neurosurgery, and bringing this software into routine use. Each section of the questionnaire was carefully designed to collect information on different aspects of the participant experience as described in Table 2. The questionnaire with the complete list of questions is reported in The questionnaire with the complete list of questions is reported in supplementary appendix questionnaire in Supplementary Material available online at doi:10.1155/2009/760364. 

Performance of participants was measured by analyzing the file containing an audit trail of the operations performed during the analysis. The performance criteria were based on the analysis settings that have major influence on the outcome of the numerical predictions, namely, the quality of the reconstructed geometry, its extension, the locations in the 1D circulation model from which boundary conditions were extracted, and the correctness of the applied boundary condition type (i.e., whether it was correctly set to inlet or outlet). Each correct operation was assigned one point, leading to a maximum score of four. Expert performance was considered as gold standard (4 out of 4) and participants performance rate was compared against this.

## 3. Results

For each section of the questionnaire only data gathered for the most representative questions were reported in this manuscript. Results were represented using tables with percentage distribution for a ready appreciation of feedback across the participants. These are reported section by section below.

**Section 1: General Feedback**

As shown in Table 3, the overall feedback about the workshop was positive. The 86% of participants would recommend the software to a colleague, 75% found the workshop useful and 78% rated their overall experience between good and very good. Negative feedback was confined averagely within less than 5%. Most participants recognized the need to improve management of IAs and for 47% this was the main reason for attending the workshop.

**Section 2: Course Design and Conduct**

80% of candidates found that the workshop to be of the right duration, 14% found that it to be too short while for 6% it was too long (Table 4). Participants-to-instructor ratio was right for 91% while 6% thought that there were too many participants. Most of the participants did not have any difficulty in understanding the instructions. On a scale of 1 to 5, where 1 is not clear and 5 is very clear, 86% rated it 4-5, while 14% were not sure. 94% of the participants thought that the course content was scientifically appropriate.

**Section 3: Experience with the Software**

34% of the candidates found the current version of the software user friendly, 11% think that it needs some improvement, while 6% found that it was not user-friendly (Table 5). The remaining 46% were unsure. 48% of the participants think that clinicians with limited IT skills will find using the software challenging, 11% disagree with this assumption and 33% were not sure. 86% of all attendees were able to complete all the steps of the hemodynamic analysis within the time allocated (approximately 50 minutes). 11% missed one or more steps. Application of boundary conditions and clipping the region of interest were among the most difficult steps reported by majority of participants. These were equally distributed among participants with scientific and clinical background.

**Section 4: Haemodynamics Understanding**

Whereas for 78% of the participants it was easy to understand the technical concepts used throughout the course (Table 6), 19% faced some difficulties in understanding the terminology, mostly related to concepts such as boundary conditions and WSS.

36% of the participants showed trust in the results predicted by the software and think that they are realistic. However, 58% were unsure. 48% believe that there is good scientific evidence to justify the role of hemodynamics in the etiopathogenesis of IAs, 3% did not agree with this. 43% of the candidates were not sure. Whereas 50% of the participants were aware of the use of CFD as a tool in the prediction of rupture in IAs, 42% were hearing the concept for the first time.

Interestingly, 84% of all participants were willing to read further peer-reviewed articles published on CFD and role of hemodynamics in IAs.

**Section 5: Impact of CFD in Neurosurgery**

Responding to the question “who should perform the CFD analysis for your patient”, 19% answered a consultant, 6% thought that it should be done by a registrar or a junior member of the team (Table 7). 25% believed that analysis should be performed by a dedicated clinical scientist/engineer, while 25% think that it can be done by anyone provided that they have adequate training.

84% of the participants were of the view that the software can be used as a diagnostic tool on outpatient basis, 8% did not agree with them. 84% of the participants were aware of similar software, whereas for 8% of them it was the first exposure to this kind of software. When asked about automated versus user controlled software, interestingly 35% expressed a wish to retain user control. 26% preferred a fully-automated tool, while 26% were unsure.

Although the majority of participants (88%) were convinced that there is a future for CFD as a risk prediction tool, and that there is a significant, or emerging clinical need for these kinds of innovative tools (84%), most of them (75%) thought that the current version of the software was not yet ready and would require refinement before it could be introduced into clinical practice.

64% of the candidates believed that an early prediction of the risk of rupture computed with the help of this software could influence their decision making in the management of an IA. Out of the 64% over half (39%) think that small asymptomatic unruptured cases specially falling in the border-line category based on current evidence, are the best cases where such software can provide definitive help. Interestingly, 19% thought that it could be useful in all cases. 69% of the participants were convinced of the need for a multicentric trial for the evaluation of the software and expressed their willingness to participate in it.

**Section 6: Bringing This Software into Routine Use**

Once the software is in routine use, 30% of the participants believed that it should be an integral part of the scanner (Table 8). 27% thought that it should be supplied as a standalone product, while 32% say it could be provided in either way.

Cost will be an important deciding factor for 72% and 81% prefer it to be a freeware or shareware. However, cost is a low priority for 17% and 11% will not mind paying for it.

**Performance**

Attendees totalized an average score of 63% of experts' performance (Table 9). When age is taken into consideration youngest delegates in the group 20–30 years scored highest (65%) with score figures reducing progressively with age. Age group 50+ obtained the lowest scores (56%). Performance was slightly higher in scientific community (2.7), as compared to the clinicians (2.5).

## 4. Discussion

### 4.1. The Current Challenges Posed by Unruptured IAs

The easy availability and widespread use of relatively noninvasive and sophisticated neurodiagnostic modalities such as high resolution CT, MRI and MRA, have brought to clinical attention a large and ever increasing, group of patients harboring unruptured and asymptomatic IAs. These unruptured lesions are also diagnosed coincidentally at the time of catheter angiography carried out for a ruptured aneurysm in patients having multiple aneurysms. The increasing awareness of relatively bleak prognosis related to aneurysmal rupture in general public and clinicians, forces neurosurgeons to come up with a definitive answer for these unruptured lesions. 

With the advancements in microsurgical techniques and improved neuroanesthetic and interventional neuroradiological approaches, the morbidity and mortality figures associated with active management of the *ruptured* IAs have improved significantly when compared to their conservative management. In other words, the indications for the active interventions in *ruptured * IAs are now well established. The situation unfortunately is not as straightforward in cases of *unruptured * IAs and, the management of these lesions remains one of the most controversial topics in Neurosurgery [1–4]. Most large series including the ISUIA studies, agree on the low risk of rupture for unruptured IAs. The cumulative rupture rates in the ISUIA studies were between 0.05 and <1 percent per annum [1, 4]. The fact that the prevalence of unruptured IAs in general population outnumbers the incidence of subarachnoid hemorrhage suggests that not all unruptured IAs share a common natural history. The annual prevalence of unruptured IAs in a population is around 5% while the incidence of subarachnoid hemorrhage in the corresponding population is observed up to a maximum of 10 cases per 100 000 persons per year [1]. It is clear from these figures that 80% to 85% of all IAs will never rupture. 

The current uncertainties in the management of unruptured IAs are well acknowledged by the clinical community, and were among the most important motivating factors for the majority of the participants (47%, Table 3) to attend this workshop. 

In order to offer the best possible treatment to the patient with the least side effects, formulation of a clear management protocol, directed by the natural history of unruptured IAs and the risks associated with the active management, is required. Whereas the endovascular coiling is increasingly being accepted as a preferred treatment modality for ruptured IAs, surgery is advocated as a first line treatment for unruptured lesions [2, 4]. Although there are no strict guidelines, most of the studies [22–24] including ISUIA trials [1, 4], almost unanimously recommend certain factors as indications of surgery in unruptured IAs, namely, large aneurysmal size, symptomatic lesions, evidence of growth, multiple lesions, posterior circulation location, and past history of SAH. All these criteria have been established to have good correlation with increased risk of rupture and hence, surgery is advocated in these situations to avoid the poor outcome. It is interesting to note that whereas on the one hand the above mentioned criteria are used to decide the need and suitability for surgery in an unruptured IA, all of these factors also remain the underlying descriptors for poor surgical outcome [25–27]. 

In the light of current evidence, it is clear that the group which will stand the best chance of an excellent outcome after surgery is the one with solitary, very small (<5 mm), truly asymptomatic IAs located in the anterior circulation, without any evidence of growth. Quite the contrary, current protocols dictate clinicians not to operate upon this group [1, 4, 25]; and in fact contraindicate any active management option in such patients [1, 4, 25]. Moreover, the small aneurysms of <5 mm size which are traditionally thought to be “safe”, are not “rupture-proof”. In a study Yasui et al. [28] found that in a group of 25 ruptured aneurysms, 16 (64%) were <5 mm in size. Similarly, Juvela et al. [29] who followed 142 patients with 181 aneurysms for a mean period of 13.9 years with an aneurysmal size of <4 mm, demonstrated a 19% rupture rate, that is, 27 out of 142 patients had a rupture. 

In order to improve the surgical outcome if we choose to operate on these smaller and “safe” lesions, we have to operate on every single patient. The ideal situation, however, would be if we could identify the aneurysms at greater risk of rupture while they are still small in size and operate upon them, leaving others to be monitored expectantly.

### 4.2. The Emerging Need for New Alternatives

It is evident that, due to the limitations associated with conventional risk factors used to assess the risk of growth and rupture, it is currently impossible to identify those patients who are at an increased risk in this subset having a real need of an early surgery from those who can be monitored safely without any active intervention. The situation consequently leaves us with no options other than searching some new descriptors which can predict the risk of rupture independently in small IAs before they join the cohort destined for a poor surgical outcome. This fact is in part reflected by the large number of participants' views (84%, Table 7), who believe that there is a significant or emerging need of new alternatives.

There is a rapidly growing body of literature affirming the importance of hemodynamics in the etiopathogenesis of IAs [5–8]. The hemodynamic variables often considered in these studies are WSS, oscillatory shear index (OSI), blood pressure and other quantities used to characterize blood flow. Proportional to blood viscosity and its velocity, WSS is the tangential frictional force exerted by the flowing blood on the walls of each vessel. High supra-physiological and low infra-physiological values of WSS have been associated with initiation, growth and rupture of aneurysms [7, 9, 11, 21, 30–33]. A measure of the oscillatory nature of these viscous forces is given by the OSI, often associated with endothelial cells degeneration [6, 15, 34]. Table 10 gives a comprehensive list of hemodynamic variables from literature and their association with IA evolution.

An evaluation of these variables can provide a useful alternative to predict the behavior of an unruptured IA at an early stage before it changes in size, shape or becomes symptomatic. Unfortunately, the detailed in vivo measurements of all relevant flow variables in the regions affected by the disease are currently impossible [9, 10].

### 4.3. Computational Fluid Dynamics: A Brief Overview

Motivated by the important role played by hemodynamics and the difficulty of conducting detailed in vivo observations of relevant hemodynamic variables, engineers and computer scientists have started using CFD to predict blood flows in IAs [9–15].

CFD is the science of predicting fluid flow, heat and mass transfer, chemical reactions, and related phenomena by solving numerically the set of mathematical equations that govern a particular physical system (conservation of mass, momentum, energy, species, etc.). Since its early development in the 1960s and 1970s in the field of aerospace, where it was used mainly to improve the design and efficiency of aircrafts [40], CFD has been successfully used in many other applications. In the past decades engineers used CFD in the automotive, nautical, and civil engineering industries for conceptual studies of new designs, troubleshooting redesign, or improving the physical understanding of a novel fluid mechanical phenomenon. Supported by experimental studies and a profound theoretical knowledge of the application at hand, CFD can be applied anywhere the flow of a fluid is important. Validation, through comparisons with experimental data, has always been a key aspect in successful applications of CFD. In the context of its use in IAs, although early validation work shows promising results [19, 41–43], a more systematic validation remains a prerequisite before CFD can be adopted as a routine tool in clinical practice. As it is evident from Table 7, 58% of the participants agreed that the results obtained using the software may influence their decision making in the small unruptured IA presented in the clinical vignette or all cases, provided they are backed by a larger clinical trial. Whereas the software at the moment can successfully predict the relevant haemodynamic indices in the context of IAs, it is expected that after the larger clinical trials, significant statistical correlations can be established forming the basis of novel clinical protocols. Whereas the majority of participants (78%, Table 6) did not find any difficulty in understanding the technical concepts used in CFD, only 36% (Table 6) of them believed that the results produced by its application were realistic. The mistrust in the results emphasises the importance of validation. This is further supported by the fact that most of the participants (84%, Table 7) readily wanted to participate in a multicentric clinical trial. 

Although participants showed a manifest interest in computational predictions (Table 6), there is a clear lack of awareness concerning the role of hemodynamics in the etiopathogenesis of IAs and the use of CFD in this context (42%, Table 6). More efforts therefore are required by the scientific community to enhance understanding of the role of hemodynamics and awareness of the use of CFD in this field.

### 4.4. The Concept of Controlled Exposure

The use of CFD in this context represents a significant change in the clinical workflow and a successful transfer of knowledge will only happen via carefully planned, controlled exposure. Clinical sites must be supported locally, underpinning the training for clinicians with the involvement of clinical scientists. The effectiveness of interdisciplinary transfer of knowledge is largely dependent on the course design and the methodology used. As reflected by the results (Table 9), a hands-on workshop using multimedia PowerPoint presentation, one-to-one supervision, and low participants-to-instructor ratio with a carefully designed course based on sound scientific principles, can lead to good results. The correct duration of such a course is also an important factor (Table 4). A close collaboration between engineers and the clinical community is also a prerequisite for successful transfer of knowledge. Supervision during this workshop was hence, jointly provided by a biomedical engineer and a clinician. Given a short training period of only 75 minute, the first ever exposure of the software and its concepts to most of the participants (Table 6), together with the fact that the software is still in its prototype stage, the overall response and average performance of 63% was remarkable. It is anticipated that performance can be enhanced to the level of the expert-user by means of a more user-friendly version of the software and more intensive training. The results also show a decline in performance with age. It may be associated with the IT skills necessary to use this type of software efficiently. This fact should be kept in mind when interpreting the results and formulating future training and translational requirements.

### 4.5. Software Design Improvement

Many valuable suggestions were collected from participants on the possible improvements in the software design and its functionalities. Among the important suggestions included automating the steps for which user intervention is not strictly necessary, improving user friendliness through a more intuitive graphical user interface (GUI) where the user is guided through the number of operations required, or use of the icons in place of the more cumbersome operation from the menu bar and, finally graphical representation of the 1D circulation model for easier application of boundary conditions. After discussing the feasibility with developers, most of these suggestions were implemented in the latest version of the software @neuFuse.

### 4.6. The Expected Place of CFD in Neurosurgery

It is interesting to note that the majority of the participants (63%, Table 7) want these analyses to be performed either by an expert clinical scientist/engineer or by a person with the same level of expertise, rather than a clinician. The fact may reflect clinicians' reluctance to conduct the analyses themselves due to their understandable concern over time-constraints and may indicate the requirement of a dedicated team with sufficient infrastructure for the purpose. In spite of this, most of the clinicians (84%, Table 7) see the software as a handy tool which can be used on an outpatient basis (e.g., ophthalmoscope, otoscope, etc.) rather than a specialist department-based facility (e.g., 3DRA, MRA, etc.). On comparing the software in terms of the different properties of a diagnostic modality which makes it an ideal outpatient tool versus those requiring a dedicated setup, we find that this software has some important features of an ideal outpatient tool. It is noninvasive and is not directly performed on the patient (patients do not have to come prepared, e.g., empty stomach). As it is totally noninvasive, there is no risk of cross-infection or contamination. Due to no associated side effects, no admission or postoperative care is necessary. Although only time will decide, in authors' view only a dedicated department with sufficient IT facilities and dedicated biomedical engineers can take the burden of the extensive computational time required by more realistic transient analyses and, effort to visualize and extract the hemodynamic characteristics required for clinical decision making.

Whereas the current study indicates a positive response among the clinical community for CFD and its use in IAs, it will be necessary to expose the software to a larger number of clinicians before definitive conclusions can be drawn.

## 5. Conclusions

Although participants showed a manifest interest in computational predictions, there is a clear lack of awareness concerning the role of hemodynamics in the etiopathogenesis of IAs and the use of CFD in this context. More efforts therefore are required by the scientific community to enhance awareness and understanding of the clinicians in the subject. There is a clear willingness to use such software as an outpatient tool. The mistrust in the results indicates the need for validations, and most of the participants supported with the need of a multicentric trial, when software is ready. Keeping in mind the very first exposure to CFD for most of the participants and the inherent difficulties associated with a developing-software, the average performance of 2.5 (63% of an expert) was remarkable. Adequate training, controlled exposure, and further development of these tools are necessary before these can be efficiently used by a common clinician. 

## Supplementary Material

Supplementary material contains questionnaire with the complete list of questions.

## Figures and Tables

**Table 1:** Aneurysm radiological characteristics.

Localization	Carotid artery/ophthalmic segment/medial wall
Side	Left
Dome status	Unruptured
Depth	4.2 mm
Max diameter	5 mm
Max neck width	3.7 mm
Type	Side-wall, saccular
Aspect	Smooth

**Table 2:** Questionnaire sections and objectives.

Section	Category	Objectives
1	General feedback	To gather impressions on the overall experience

2	Course design and conduct	To gather suggestions on possible improvements and identify any shortcomings in the design of the workshop

3	Experience with the software	To identify weak points as perceived by clinicians in the graphical user interface of the current version of the software

4	Haemodynamics understanding	To assess their current knowledge and understanding in the role of haemodynamics in the aetiopathogenesis of intracranial aneurysms

5	Impact of CFD in neurosurgery	To assess their faith in the principles of CFD and its use in the clinical environment, along with the need of validation through a multicentre trial

6	Bringing this software into routine use	To explore the participants view on cost related issues and gather information on future marketing potentials of this kind of software

**Table 3:** General feedback.

*Question*/Answer options	Number of participants (%)	
*Would you recommend the software to a colleague?*		
Yes	31	(86)
No	3	(8)
n.a.	2	(6)

*Why did you decide to participate to this workshop?*		
Working in the field	16	(45)
Interested in CFD	2	(6)
Improve management of aneurysms	17	(47)
Other	1	(2)

*How useful did you find this workshop?*		
Not useful	1	(3)
Not sure	8	(22)
Useful	16	(45)
Very useful	11	(30)

*Rate your overall experience*		
Poor	1	(3)
Average	7	(19)
Good	15	(42)
Very good	13	(36)

**Table 4:** Course design and conduct.

*Question*/Answers	Number of participants (%)	
*Was the duration of the workshop*…		
Right	29	(80)
Short	5	(14)
Long	2	(6)

*Was the participant-to-instructor ratio*…		
Right	33	(91)
Too-many	2	(6)
n.a.	1	(3)

*Were the instructions given in a clear way?*		
Not sure	5	(14)
Clear	18	(50)
Very clear	13	(36)

* Was the content of the course scientifically appropriate?*		
Yes	34	(94)
No	2	(6)

**Table 5:** Experience with the software.

*Question*/Answers	Number of participants (%)	
*Do you find the software user friendly?*		
No	2	(6)
Needs improvement	4	(11)
Not sure	17	(46)
User friendly	11	(31)
Very user friendly	1	(3)
n.a.	1	(3)

*Will clinicians without tech/IT experience have trouble?*		
Yes	17	(48)
Not sure	12	(33)
No	4	(11)
n.a.	3	(8)

*Were you able to complete all the steps of the hemodynamic analysis?*		
Yes	30	(86)
No	4	(11)
n.a.	1	(3)

**Table 6:** Haemodynamics understanding.

*Question*/Answers	Number of participants (%)	
*Did you have difficulty with the technical concepts (boundary conditions, wall shear stress, etc.)?*		
Yes	7	(19)
No	28	(78)
n.a.	1	(3)

*Are the results from this software realistic?*		
Yes	13	(36)
Not sure	21	(58)
No	0	(0)
n.a.	2	(6)

*Is current evidence sufficient to justify a role for haemodynamics in the pathogenesis of aneurysms?*		
No	1	(3)
Not sure	15	(43)
Yes	17	(48)
n.a.	2	6

*Were you previously aware of the use of CFD to predict the risk of rupture in intracranial aneurysms?*		
No	15	(42)
Yes	18	(50)
n.a.	3	(8)

*If you see a publication on computational predictions for IA in a peer-reviewed journal, will you read it?*		
No	3	(8)
Yes	30	(84)
n.a.	3	(8)

**Table 7:** Impact of CFD in neurosurgery.

*Question*/Answers	Number of participants (%)	
*Ideally, who should perform this type of computational analysis for patients?*		
Consultant	7	(19)
Dedicated clinical scientist	9	(25)
Registrar	2	(6)
Anyone with training	14	(38)
Office member	1	(3)
All	1	(3)
n.a.	2	(6)

*When ready could this software be used diagnostically in an outpatient clinic?*		
Yes	30	(84)
No	3	(8)
n.a.	3	(8)

*Are you aware of any similar software?*		
Yes	30	(84)
No	3	(8)
n.a.	3	(8)

*Should this type of analysis be fully automated, or is it better that the user has control?*		
Automated	10	(26)
User control	14	(35)
Not sure	10	(26)
n.a.	5	(13)

*Is there a future for computational tools for risk prediction of intracranial aneurysm rupture?*		
Yes	29	(80)
No	2	(6)
Not sure	2	(6)
n.a.	3	(8)

*How great a clinical need is there for this software?*		
Significant	17	(48)
Emerging	13	(36)
Low	3	(8)
n.a.	3	(8)

*Do you think that this type of analytical software is ready for introduction into the clinical environment?*		
Ready	4	(11)
Needs work	26	(75)
n.a.	5	(14)

*In which cases might this software influence your decision-making about patient management?*		
All	7	(19)
Small unruptured asymptomatic	14	(39)
Other	2	(6)
Not sure	1	(3)
None	3	(8)
n.a.	9	(25)

*Would you be interested in participating in a multicentre trial on the evaluation of this software?*		
Yes	25	(69)
No	5	(14)
n.a.	6	(17)

**Table 8:** Bringing this software into routine use.

*Question*/Answers	Number of participants (%)	
*Would you expect this software to be provided as part of a scanner, or as a stand-alone product?*		
Scanner	11	(30)
Standalone	10	(27)
Both	12	(32)
n.a.	4	(11)

*Would the price of this software be an important factor in your deciding to obtain/use it?*		
Important	26	(72)
Low priority	6	(17)
n.a.	4	(11)

*Would you expect to pay for this software, or would you prefer a freeware/shareware arrangement?*		
Pay	4	(11)
Shareware	14	(37)
Freeware	17	(44)
n.a.	3	(8)

**Table 9:** Attendees' performance.

	Score	%
*Average*	2.52	(63)

*Performance with age*		
20–30 years	2.58	(65)
31–40 years	2.50	(63)
41–50 years	2.36	(59)
50+ years	2.22	(56)

*Performance with background*		
Clinicians	2.5	(63)
Scientists	2.7	(68)

**Table 10:** Literature-based evidence on the importance of hemodynamics in the etiopathogenesis of ICAs. NB: WSS; wall shear stress, MMP-13; matrixmetalloproteneases-13, iNOS; inducible-nitric oxide synthase, NO; nitric oxide, OSI; oscillatory shear index.

Hemodynamic factors	Intracranial aneurysm			Proposed mechanism(s)	References
	Initiation	Growth	Rupture		
*Dynamic*					
Wall shear stress (WSS)	High	Low	Low	Increased WSS increases the production of MMP-13 which in turn leads to vessel wall damage. Decreased WSS increases iNOS synthesis—NO induced damage to vessel wall. Low WSS increases endothelial proliferation and apoptosis	Boussel et al. [11], Fukuda et al. [30], Gao et al. [7], Jou et al. [31], Malek et al. [21], Meng et al. [32], Shojima et al. [9], Ujiie et al. [33]

Oscillatory shear index (OSI)	High/Low	High	High	Degenerative changes in endothelium	Glor et al. [35], Goubergrits et al. [34], Mantha et al. [15]

Jet of blood stream	Impingement	Impingement	Impingement	Localized endothelial cell injury	Foutrakis et al. [36], Cebral et al. [14], Cebral et al. [37]

Flow pattern	—	—	Complex	Statistical association	Cebral et al. [14, 37]

*Hydrostatic*					
Pressure	High	High	High	Passive yield/water hammer effect	Inci and Spetzler [38], Morimoto et al. [8] Steiger et al. [39]
