# Supplementary material for: The Role of Computational Fluid Dynamics in the Management of Unruptured Intracranial Aneurysms: A Clinicians' View
Source: Comput Intell Neurosci. 2009 Aug 19;2009:760364. doi: 10.1155/2009/760364 (PMC2729101; doi:10.1155/2009/760364)
Supplement: Supplementary file 1 — Supplementary material contains questionnaire with the complete list of questions. [file 760364.f1.doc]

#
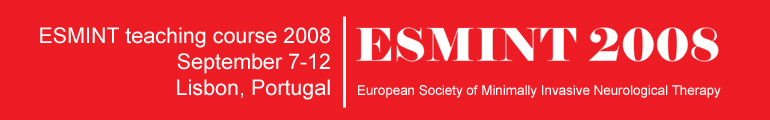

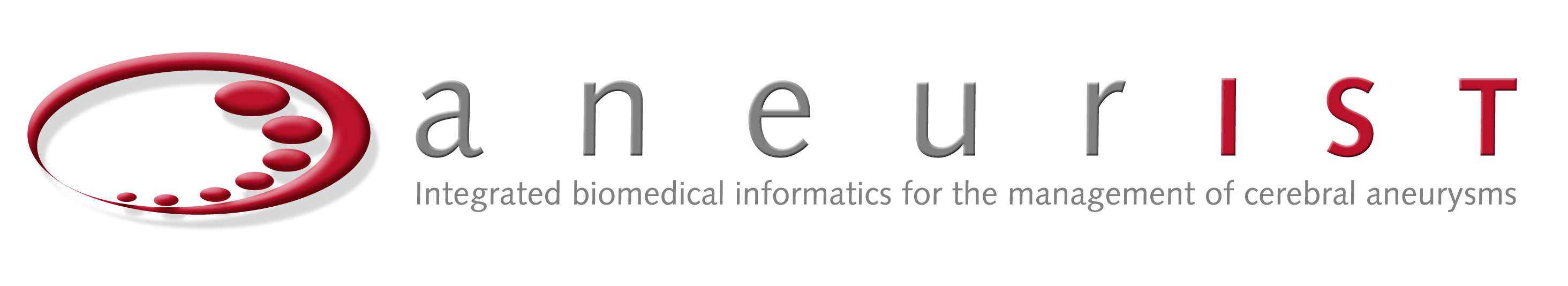


# Edificio “Egas Moniz”, Hospital de Santa Maria Faculdate de Medicina da Universidade de Av. Professor Egas Moniz 1649-028 Lisboa, Portugal <http://www.fm.ul.pt/>

# Workshop Evaluation - @neuFuse

### Personal Details

| Title: | First Name: Surname: Age: |
| --- | --- |
| Degree: |  |
| Job Title: |  |
| Institution/Department: |  |
| Background: | Clinical Engineering Scientific Other: |
| Address: |  |
| Email: |  |
| Telephone: |  |

### Section 1: General Feedback

| 1. Why did you decide to participate to this workshop? |  Working in the field   Suggestion by colleague   Interested in computational haemodynamics   Improve management of aneurysms   Other: please specify  ……………………………………………. |
| --- | --- |
| 1. How useful did you find this workshop? | Not 1 2 3 4 5 Very |
| 1. Would you recommend a friend to attend? |  No  Yes |
| 1. Would you recommend the software to a friend |  No  Yes |
| 1. Any specific shortcomings, surprises? | 1.  2.  3. |
| 1. Any suggestions for course improvements? |  No  Yes, I’ve described them below  ……………………………………………. |
| 1. Rate your overall experience… | Bad 1 2 3 4 5Good |

### Section 2: Course Design and Conduct

| 1. Was the participant-to-instructor ratio… |  OK  Too many students |
| --- | --- |
| 1. Were the instructions given in a clear way? | No 1 2 3 4 5 Very |
| 1. Was the content of the course scientifically appropriate? |  No  Yes |
| 1. Do you think the instructors were helpful? | No 1 2 3 4 5Very |
| 1. How useful was the presentation and notes? | Not 1 2 3 4 5Very |
| 1. Did you have any difficulty with terminology? |  No  Yes, I’ve described it below  ……………………………………………. |
| 1. Were the IT facilities satisfactory? | No 1 2 3 4 5Very |
| 1. Was the duration of the workshop… | Too short Just right Too long |

### Section 3: Experience with the Software

| 1. Do you find the software user-friendly? | No 1 2 3 4 5 Very |
| --- | --- |
| 1. Will clinicians without tech/IT experience have trouble? |  No  Not Sure  Yes |
| 1. Were you able to complete all the steps of the haemodynamic analysis? |  No, I missed those below  Yes  ……………………………..…………. |
| 1. Is there any obvious limitation which may prevent the use of this software in future? |  No  Yes, I’ve described it below  ……………………………………………. |
| 1. Please, identify the easiest and most difficult steps in the use of @neuFuse | Easiest: ……………..………..  Most Difficult: ………………………. |
| 1. What would you change/improve in the software? | 1.  2.  3. |
| 1. In particular, do you think that the Graphical User Interface (GUI) can be improved in any way? |  No  Yes, I’ve described it below  …………………………………………. |
| 1. Do you now feel confident in the use of this software? | No 1 2 3 4 5 Very |
| 1. Would you like to be kept informed about developments of this software? |  No  Yes |

### Section 4: Hemodynamic Understanding

| 1. Did you have difficulty with the technical concepts (boundary conditions, wall shear stress, etc.)? |  No  Yes, I’ve named them below  ……………………………………………. |
| --- | --- |
| 1. Are the results from this software realistic? |  No  Yes  Not sure |
| 1. Is current evidence sufficient to justify a role for haemodynamics in the pathogenesis of aneurysms? |  No  Yes  Not sure |
| 1. Were you previously aware of the use of CFD to predict the risk of rupture in intracranial aneurysms? |  No  Yes |
| 1. If you see a publication on computational predictions for IA in a peer-reviewed journal, will you read it? |  No  Yes |
| 1. Would you be interested in receiving high-quality peer-reviewed publications on haemodynamics in IA? |  No  Yes |

### Section 5: Impact of CFD in Neurosurgery

| 1. Ideally, who should perform this type of computational analysis for patients? |  Consultant   A dedicated clinical scientist/ engineer   Registrar/ junior member of team   Anyone provided with adequate training   It should be simplified so it’s an office job   Other, please specify:  ……………………………………………. |
| --- | --- |
| 1. Could this software be used diagnostically in an outpatient clinic? |  No  Yes |
| 1. Are you aware of any similar software? |  No  Yes, I’ve named it below  ……………………………………………. |
| 1. Should this type of analysis be fully automated, or is it better that the user has control? |  Automate  Not Sure User control |
| 1. Is there a future for computational tools for risk prediction of intracranial aneurysm rupture? |  No  Yes  Not sure |
| 1. How great a clinical need is there for this software? |  Significant  Low  Emerging |
| 1. Do you think that this type of analytical software is ready for introduction into the clinical environment? |  Ready  Not required  Needs work |
| 1. In which cases might this software influence your decision-making about patient management? |  None  All  Those below…  ……………………………………………. |
| 1. Would you be interested in participating in a multi-centre trial on the evaluation of this software? |  No  Yes |
| 1. Can you see any legal or ethical implications in the use of this software? |  No  Yes, I’ve described them below  ……………………………………………. |
| 1. The principles employed in @neurIST are applicable to other diseases; is this important? |  Important  Only care about IA |
| 1. More complex and informative analyses take longer to calculate; are quick results better? |  Quick  Complex  Let me choose |
| 1. @neurIST IA rupture risk assessment can use more that haemodynamic data; is this important? |  Important  Only need haemodynamics |
| 1. @neurIST offers tools for researchers as well as clinicians; are these important? |  Important  Only need clinical |

### Section 6: Bringing this Software into Routine Use

| 1. Would you expect this software to be provided as part of a scanner, or as a stand-alone product? |  Scanner  Standalone  Both |
| --- | --- |
| 1. Would you expect such software to be provided by a scanner company or an independent specialist? |  Scanner Co  Independent |
| 1. Would you expect to pay for this software, or would you prefer a freeware/shareware arrangement? |  Pay  Freeware  Shareware |
| 1. Would the price of this software be an important factor in your decision to obtain/use it? |  Important  Cost is a lower priority |

### Any other comments?

***Thank-you for completing this Questionnaire!***

OFFICE USE ONLY

### Instructor’s evaluation of individual’s performance

| Performance | 1 2 3 4 |
| --- | --- |
